# Physicochemical and Antioxidant Properties of Black Garlic

**DOI:** 10.3390/molecules191016811

**Published:** 2014-10-20

**Authors:** Il Sook Choi, Han Sam Cha, Young Soon Lee

**Affiliations:** Department of Food and Nutrition, Kyung Hee University, 1 Hoeki-dong, Dongdaemun-Ku, Seoul 130-701, Korea; E-Mails: choiis02@khu.ac.kr (I.S.C.); abcing02@gmail.com (H.S.C.)

**Keywords:** black garlic, aging period, physicochemical characteristics, antioxidant contents, antioxidant activities

## Abstract

Black garlic (BG) is a processed garlic product prepared by heat treatment of whole garlic bulbs (*Allium sativum* L.) at high temperature under high humidity for several days, resulting in black cloves with a sweet taste. BG has recently been introduced to the Korean market as a product beneficial to health. To clarify how BG changes during the 35 day aging period, the physicochemical characteristics, antioxidant contents, and antioxidant activities were evaluated under controlled conditions of 70 °C and 90% relative humidity. Reducing sugar and total acidity of BG increased during the aging period, whereas pH decreased from pH 6.33 to 3.74. Lightness and yellowness values of BG radically decreased during the aging period, whereas redness values increased significantly. Antioxidant components, including the total polyphenol and total flavonoids contents of BG, increased significantly until the 21st day of aging (*p* < 0.05) and correspondingly, the antioxidant activities of BG, measured by DPPH, ABTS, FRAP, and reducing power assays, were highest on the 21st day of aging. These results indicate that BG can be considered to not only possess antioxidant properties during the aging period, but also to reach its optimal antioxidant properties at the 21st day of aging.

## 1. Introduction

Garlic (*Allium sativum* L.), belonging to the Alliaceae family, is a frequently used ingredient in gastronomy. Garlic has also been used as a traditional medicine for a variety of biological effects, such as increasing stamina, aiding digestion to prevent diarrhea and worm infestation, and treating heart disease, arthritis, and fatigue [1]. Recently, numerous studies have shown garlic to possess a wide range of bioactive effects, including antioxidant, antimicrobial, anticancer, antihypertensive, hepatoprotective, and insecticidal properties [2]. Although the bioactive properties of garlic are related to antioxidant polyphenolic and bioactive sulfur compounds [3], when garlic is crushed or damaged, some of these bioactive sulfur components produce a strong pungent odor, which are associated with an unpleasant body and breath odor in consumers. The garlic preparation processes are important factors when choosing a garlic supplement because of the various biologically active compounds of garlic present and consumer acceptance. Several garlic products, such as dehydrated garlic powder, garlic essential oil, garlic oil macerate, and aged garlic extract have been introduced to the market and are currently available [4].

Black garlic (BG) has been recently introduced to the Korean market as a health product. BG is formed by aging whole garlic at high temperature and in high humidity, causing the garlic to turn black because of browning compounds. Furthermore, BG does not exude a strong off-flavor, like fresh garlic. This is because of changes to the compound allicin, which is responsible for the pungent odor, into water soluble antioxidant compounds such as *S*-allylcysteine, tetrahydro-β-carbolines, biologically active alkaloids, and flavonoid-like compounds [3,5]. S-Allylcysteine is formed by the catabolism of γ-glutamylcysteine and it inhibits oxidative damage related with aging and various diseases [6]. Tetrahydro-β-carboline derivatives, which have been identified in BG extracts, also show antioxidant effects [7,8]. Tetrahydro-β-carboline derivatives are formed by condensation between tryptophan and aldehyde, similar to the production of pyruvic acid by the allin-allicin pathway or the Maillard reaction process [5]. Furthermore, several studies have reported that BG extracts have antioxidative, anti-allergic, anti-diabetic, anti-inflammatory, hypocholesterolemic, hypolipidemic, and anti-carcinogenic effects [5,6,7,8,9,10,11,12,13,14]. However, these studies have used various aging conditions, ranging from 4 to 40 days. Therefore, we hypothesized that there should be optimum aging conditions to maximize the antioxidant properties of BG. Our objective of this study was thus to identify the physicochemical properties of BG during 35 days of aging, and to identify the optimum aging period for maximized antioxidant properties. To achieve this, we quantified the bioactive compound levels, including total polyphenols and flavonoids contents, and antioxidant activities of BG, using 1,1-diphenyl-2-picryl hydrazyl (DPPH), 2,2-azino-bis-(3-ethylbenozothiazoline-6-sulfonic acid) (ABTS), ferric reducing antioxidant power (FRAP), and reducing power assays.

## 2. Results and Discussion

### 2.1. Physicochemical Properties of Black Garlic

The physicochemical characteristics of BG during the aging period are presented in Table 1. The total acid content of BG increased significantly compared to that of raw garlic, while the pH of BG significantly decreased from 5.49 to 3.74, when compared to 6.33 in raw garlic, during the aging period (*p* < 0.05). This result is in agreement with the report of Shin *et al.* [15], which showed that the pH of BG decreased from 6.40 to 5.29 after 6 days of aging. The reducing sugar content of BG increased approximately 6-fold, from 2.73 g/kg on the 7th day to 16.07 g/kg on the 35th day, and these values were significantly higher than those of raw garlic (1.52 g/kg). This result is in agreement with the data of Choi *et al.* [16], which show that sugar content (e.g., glucose, fructose, sucrose, and maltose) increased in BG compared to fresh and steamed garlic. Furthermore, this increased sugar content of BG might be related to its sweet taste.

**Figure 1:**
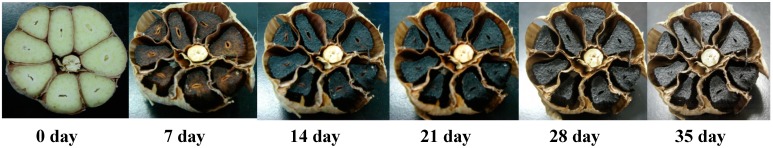
Changes in the color of black garlic during the aging period.

**Figure 2:**
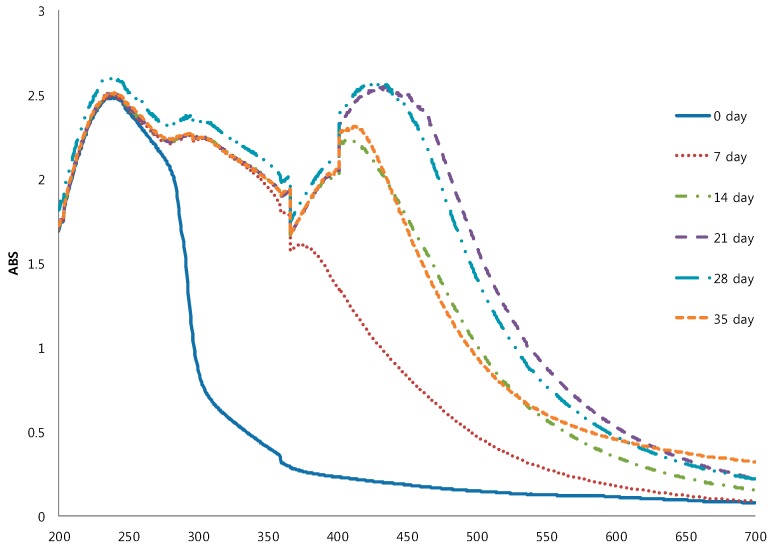
Visible spectra of black garlic during the aging period.

Color is one of the most important psychological properties of food products that affect the perception of eating in consumers. The color of BG changed to dark brown during the aging period (Figure 1, Table 1). Color redness (a* value) of BG dramatically increased during the aging period, while lightness (L* value) and yellowness (b* value) decreased relative to raw garlic. Next, we focused on the impact of BG color development using UV-visible spectroscopy. Figure 2 shows the difference in spectral patterns between raw garlic and BG during 35 days of aging. Raw garlic showed a maximum absorbance at around 230 nm, while BG, on the 7th day, showed a maximum absorbance at 230 nm, around 280–310 nm, and also slightly at 360 nm. However, on the 14th and 35th day, BG showed additional shoulders around 400–425 nm, whereas on the 21st day and the 28th day, BG showed maximum absorbance in the visible 425–450 nm range. Color changes resulting from heat treatment are typically due to the Maillard reaction, known as non-enzymatic browning reaction. According to the property of the reactants involved, the Maillard reaction products are usually associated with the absorbance increases at 280 nm, 320–360 nm, and 420–450 nm, corresponding to the initial, intermediate, and final stages of Maillard reaction products (MRPS) formation, respectively [17]. The initial stage of the Maillard reaction produces colorless (about 280 nm) intermediates arising from sugar-amine condensation and amadori rearrangement. The intermediate stage (320–360 nm) produces colorless or yellow products via several reactions, such as sugar dehydration, sugar fragmentation, and amino acid degradation (Strecker degradation). The final stage (420–450 nm) is highly colored with aldol condensation, aldehyde-amine condensation, and formation of heterocyclic nitro compounds. Billaud *et al.* [18] showed that MRPs derived from glucose and cysteine had one absorption shoulder at 285 nm with a maximum absorbance at 340–360 nm. The formation of MRPs depends on the processing conditions such as temperature and time [19]. Therefore, color changes of black garlic, such as increasing redness and decreasing lightness and yellowness, may be related to the formation of MRPs during the aging period at 70 °C.

**Table 1:** Physicochemical characteristics of black garlic during aging period.

Components	Aging Period (Days)					
	0	7	14	21	28	35
**Moisture (%)**	64.21 ± 1.48 ^a^	32.72 ± 0.97 ^b^	31.77 ± 2.60 ^b^	31.12 ± 0.17 ^b,c^	29.55 ± 0.39 ^c^	29.88 ± 0.49 ^c^
**Total acidity (mg/kg)**	0.40 ± 0.01 ^e^	1.30 ± 0.01 ^d,e^	1.50 ± 0.02 ^c,d^	1.70 ± 0.03 ^c^	2.30 ± 0.06 ^b^	2.60 ± 0.03 ^a^
**pH**	6.33 ± 0.07 ^e^	5.49 ± 0.09 ^d,e^	4.41 ± 0.17 ^c,d^	4.22 ± 0.08 ^c^	4.07 ± 0.02 ^b^	3.74 ± 0.062 ^a^
**Reducing sugar (g/kg)**	1.52 ± 0.01 ^d^	2.73 ± 0.32 ^c^	12.42 ± 0.85 ^b^	15.96 ± 0.29 ^a^	15.98 ± 0.23 ^a^	16.07 ± 0.38 ^a^
**Color**						
L*	68.44 ± 1.66 ^a^	15.67 ± 2.41 ^b^	9.28 ± 1.74 ^c^	5.61 ± 0.68 ^d^	5.19 ± 1.11 ^d^	4.33 ± 2.02 ^d^
a*	−3.84 ± 0.46 ^c^	6.13 ± 0.95 ^a^	5.45 ± 0.94 ^a^	5.23 ± 1.06 ^a^	3.18 ± 1.46 ^b^	2.73 ± 1.01 ^b^
b*	26.59 ± 1.76 ^a^	10.65 ± 4.16 ^b^	2.37 ± 7.47 ^c^	−3.07 ± 4.60 ^d^	−3.76 ± 3.59 ^d^	−3.86 ± 1.49 ^d^

Values are mean ± SD; Values followed by different letters in the same row are significantly different by Duncan’s multiple range test (*p* < 0.05).

### 2.2. Amino Acid Changes of Black Garlic

Changes in the free amino acids of BG during the aging period are shown in Table 2. Contents of branched amino acids (leucine and isoleucine) were higher in BG than those in raw garlic. Cysteine is an important precursor of sulfur-containing compounds in garlic, such as *S*-methyl-l-cysteine sulphoxide (methiin) and *S*-allyl-l-cysteine sulphoxide (alline), which are the most abundant and the parent cysteine-containing compound responsible for the odoriferous compounds [20]. After processing steps such as cutting, crushing, or dehydration, these compounds are decomposed into other volatile compounds, including diallyl sulfide, diallyl disulfide, diallyl trisulfide, dithirne, and ajocene [3]. Here we found that the sulfur-containing cysteine content of BG decreased significantly during aging and that the cysteine content of BG was lower than that of raw garlic. These results might be associated with the reduced sulfurous flavor of BG. Interestingly, among aromatic amino acids, the phenylalanine content (82.38 to 143.07 mg/100 g) of BG increased during aging and the content was higher than that of raw garlic (55.64 mg/100 g), whereas the tyrosine content (77.31 to 109.13 mg/100 g) of BG decreased dramatically during aging compared to that of raw garlic (449.95 mg/100 g). Hwang *et al.* [21] reported the MRPs of the sugar-cysteine and sugar-tyrosine model systems were higher in antioxidant activity than those of other MRPs. The decrease of cysteine and tyrosine contents of BG during aging period could be related to the changes of antioxidant activity of BG. Acidic amino acid contents of tyrosine and aspartic acid, and basic amino acid contents of glutamic acid, arginine, and lysine decreased as aging period continued. Likewise, the contents of polar amino acids like threonine and serine, and nonpolar amino acids like glycine and alanine decreased compared to the raw garlic. Therefore, the decrease of these amino acids, especially cysteine and tyrosine, during the aging period might be related to Maillard reactions, which occur between amines which are usually amino acids and carbonyl compounds which are usually reducing sugars.

**Table 2:** Amino acid contents of black garlic during the aging period.

Amino Acid (mg/100g)	Aging Periods (Days)					
	0	7	14	21	28	35
**Leusine**	58.62 ± 3.37 ^b^	73.44 ± 1.79 ^a^	62.81 ± 1.51 ^b^	62.12 ± 5.64 ^b^	61.23 ± 4.32 ^b^	59.19 ± 3.94 ^b^
**Isoleucine**	50.04 ± 13.47 ^b^	89.25 ± 18.93 ^a^	86.45 ± 8.23 ^a^	83.79 ± 2.37 ^a^	79.44 ± 1.14 ^a^	71.07 ± 2.25 ^a^
**Valline**	47.74 ± 0.19 ^a^	47.68 ± 0.64 ^a^	36.71 ± 4.64 ^b^	35.23 ± 5.61 ^b^	34.73 ± 8.61 ^b^	33.91 ± 6.61 ^b^
**Methionine**	31.56 ± 1.40 ^b^	82.51 ± 4.70 ^a^	80.73 ± 6.37 ^a^	78.11 ± 2.33 ^a^	73.59 ± 9.71 ^a^	71.11 ± 3.55 ^a^
**Cysteine**	81.06 ± 0.95 ^a^	69.43 ± 0.94 ^b^	49.20 ± 5.42 ^c^	46.90 ± 8.98 ^c^	43.44 ± 3.38 ^c^	42.14 ± 7.18 ^c^
**Phenylalanine**	55.64 ± 0.74 ^c^	82.38 ± 8.35 ^b^	70.20 ± 4.41 ^b^	135.16 ± 7.10 ^a^	136.25 ± 12.76 ^a^	143.07 ± 6.32 ^a^
**Tyrosine**	449.95 ± 6.29 ^a^	109.13 ± 26.09 ^b^	102.33 ± 0.38 ^b^	82.28 ± 7.41 ^b^	78.35 ± 4.34 ^b^	77.31 ± 7.54 ^b^
**Aspartic acid**	90.12 ± 2.55^ b^	117.50 ± 9.07 ^a^	64.53 ± 5.84 ^c^	62.43 ± 4.34 ^c^	61.65 ± 9.12 ^c^	60.19 ± 8.16 ^c^
**Glutamic acid**	286.60 ± 8.09 ^a^	128.87 ± 9.09 ^b^	112.81 ± 3.02 ^b^	108.11 ± 9.12 ^b^	101.88 ± 7.71 ^b^	100.11 ± 6.09 ^b^
**Arginine**	409.05 ± 3.00 ^a^	340.20 ± 75.68 ^b^	119.30 ± 0.52 ^d^	208.71 ± 51.36 ^c^	71.92 ± 0.65 ^d,e^	40.34 ± 1.10 ^e^
**Lysine**	61.68 ± 5.29 ^a^	57.96 ± 4.93 ^a^	47.39 ± 2.24 ^b^	42.50 ± 2.27 ^b^	41.53 ± 3.67 ^b^	40.50 ± 9.22 ^b^
**Histidine**	89.12 ± 1.88 ^b^	191.69 ± 18.89 ^a^	58.76 ± 13.02 ^c^	58.75 ± 3.59 ^c^	57.62 ± 5.51 ^c^	57.89 ± 8.34 ^c^
**Threonine**	81.25 ± 13.59 ^a^	46.30 ± 0.87 ^b^	53.53 ± 8.36 ^b^	57.54 ± 2.22 ^b^	58.36 ± 11.17 ^b^	59.36 ± 5.22 ^b^
**Serine**	38.53 ± 0.82 ^a^	25.72 ± 0.87 ^b^	25.84 ± 0.33 ^b^	24.78 ± 1.12 ^b^	24.23 ± 4.22 ^b^	23.71 ± 5.41 ^b^
**Glycine**	21.50 ± 2.51 ^b^	37.41 ± 8.54 ^a^	9.60 ± 1.78 ^c^	8.63 ± 1.18 ^c^	ND	ND
**Alanine**	89.72 ± 11.44 ^b^	239.13 ± 17.84 ^a^	67.01 ± 2.39 ^c^	83.59 ± 5.71 ^b^	47.38 ± 1.74 ^d^	32.74 ± 5.37 ^d^

Values are mean ± SD; Values followed by different letters in the same row are significantly different by Duncan’s multiple range test (*p* < 0.05). ND, cannot be detected.

### 2.3. Antioxidant Content of Black Garlic

To clarify the antioxidant properties of BG during aging, we focused on the analysis of total polyphenol and total flavonoids contents (Table 3). The total polyphenol contents (25.81–58.33 mg GAE/g) of BG were not only significantly higher than those of raw garlic (13.91 mg GAE/g) but also increased significantly until the 21th day of aging, before decreasing during the rest of the aging period (*p* < 0.05). According to Kim *et al.* [22], hydroxycinnamic acid derivatives and other phenolic acid contents are increased over 5-fold in black garlic compared to raw garlic. These increases of phenolic acids might also be related to an increase of the total acid contents of BG. According to Xu *et al.* [23], heat treatment of the phenolic compounds increased the free fraction of phenolic acids, whereas it decreased the ester, glycoside, and ester-bound fractions, leading to an increase in free phenol forms. Gorinstein *et al.* [24] showed that the garlic processing conditions lead to changes in the contents of its bioactive compounds, such as polyphenols, flavonoids, and anthocyanins, and that this is connected to the type and duration of treatment. Flavonoids do not only belong to a group of variable phenolic structures but are also found in fruit, vegetables, grains, roots, flowers, tea, and wines [25]. Heat treatment has a large influence on flavonoid availability, dependent on the magnitude and duration of treatment, their sensitivity to heat, and the physicochemical food environment [26]. During heat treatment, total flavonoids content increases and decreases in food products depending on the processing conditions [26]. The total flavonoid content of BG (5.38 mg RE/g to 16.26 mg RE/g) was not only significantly higher than that of raw garlic (3.22 mg RE/g), but also increased significantly up to the 21st day of aging, after which the continued to increase slightly for the remainder of the aging period (*p* < 0.05). From the results of total polyphenols, and total flavonoids, the optimum aging period of BG to maximize antioxidant content may be the 21st day of aging.

**Table 3:** Antioxidant contents of black garlic during aging period.

	Aging Period (Days)					
	0	7	14	21	28	35
Total polyphenol (mg GAE/g)	13.91 ± 1.62 ^f^	25.81 ± 1.59 ^e^	35.28 ± 0.32 ^d^	58.33 ± 1.90 ^a^	55.25 ± 0.70 ^b^	48.35 ± 1.14 ^c^
Total flavonoid (mg RE/g)	3.22 ± 0.07 ^d^	5.38 ± 0.06 ^c^	8.34 ± 0.61 ^b^	15.37 ± 0.52 ^a^	16.26 ± 1.69 ^a^	15.70 ± 2.11 ^a^

GAE = gallic acid equivalents; RE = rutin equivalents; Values are mean ± SD; Values followed by different letters in the same row are significantly different by Duncan’s multiple range test (*p* < 0.05).

### 2.4. Antioxidant Activity of Black Garlic

**Figure 3:**
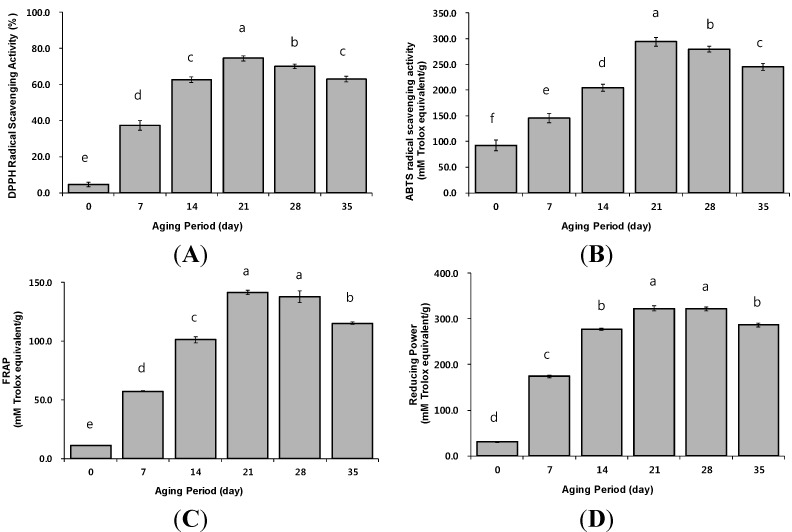
Antioxidant activities of black garlic during the aging period. (**A**) DPPH radical scavenging; (**B**) ABTS radical scavenging; (**C**) Fe(III) reducing (pH 3.6); (**D**) Fe(III) reducing (pH 6.6). Different letters above the bars for the same aging periods indicate significant difference at *p* < 0.05 (Duncan’s test).

To accurately assess the antioxidant activities of BG, four distinct antioxidant activity measurements were performed. DPPH radical scavenging activity and ABTS assays are characterized by the ability of the corresponding substrates to undergo single electron transfer by two components in the reaction mixture of antioxidants with oxidant, such as the DPPH and ABTS radicals, respectively. The assays are also operationally simple and easy to use [27]. The DPPH free radical scavenging activity of BG (37.32%–74.48%) was significantly higher than that of raw garlic (4.65%) (Figure 3A). The DPPH free radical scavenging activity of BG increased approximately 2-fold, from 37.32% on the 7th day to 74.48% on the 21st day, and then slightly decreased to 63.09% till the 35th day of aging. The values were significantly higher than that of raw garlic (4.65%) (*p* < 0.05). This pattern of DPPH free radical activity of BG was similar to that of the total polyphenol content (Table 3). The ABTS•+ free radical scavenging activity of BG was significantly higher on the 21st day (249.20 mM TE), compared to the raw garlic (92.43 mM TE). The values steadily decreased from the 21st day to the 35th day (245.45 mM TE) (Figure 3B). These results were similar to the DPPH free radical scavenging effects. Generally, the FRAP assay and reducing power are based on the ability of an electron transfer reaction, with a ferric salt as oxidant. The FRAP assay requires acidic conditions (pH 3.8), whereas the reducing power assay occurs at neutral pH [27]. As shown in Figure 3C, the FRAP values of BG were dramatically higher than those of raw garlic. Like in the other assays, the FRAP value of BG increased up to the 21st day of aging and then subsequently decreased. During the aging period, the antioxidant contents (e.g., total polyphenol, total flavonoids) of BG gradually increased up to the 21st day (Table 3), which is likely associated with the antioxidant results observed in the FRAP assay. Furthermore, the reducing power of BG also dramatically increased up to the 21st day (322.70 mM TE) when compared to raw garlic (30.55 mM TE) (Figure 3D). These results were similar to those obtained in the DPPH, ABTS, and FRAP assays and the increase in the antioxidant activities of BG may be due to the increase in total polyphenols, total flavonoids, and ascorbic acid contents during aging period. Based on the above results of antioxidant compounds and antioxidant activities findings, we propose that the optimum aging period for maximizing the antioxidant properties of BG is 21 days.

## 3. Experimental Section

### 3.1. Materials and Reagents

Fresh garlic (*Allium sativum* L.) was purchased from a local market in the Uiseong Agricultural Association, Gyeongsangbuk-do, South Korea in 2011. Folin-Ciocalteu’s reagent, garlic acid, rutin, 1,1-diphenyl-2-picrylhydrazyl (DPPH), 2,2'-azino-bis-(3-ethylbenozothiazoline-6-sulfonic acid) (ABTS), potassium persulphate, trichloroacetic acid (TCA), and potassium ferricynide were purchased from Sigma-Aldrich Chemical Co. (St. Louis, MO, USA). All chemicals and reagents used in this study were of analytical grade.

### 3.2. Preparation and Extraction of Black Garlic Extract

BG samples were prepared based on the method described in [8]. Ten unpeeled raw garlic heads were incubated in a thermohygrostatic chamber (THPE 025, Jeio Tech, Seoul, Korea) at 70 °C in 90% relative humidity for 7, 14, 21, 28, and 35 days. During the aging process, raw garlic developed a black color. The fresh raw garlic and aged BG cloves were peeled from the bulb and ground by a high-speed mixer (Blaender 7012S, Waring, Torrington, CT, USA). The ground BG was mixed with deionized water at a solid:liquid ratio of 1:3. The samples were extracted three times with deionized water for 1 h at room temperature in a shaker (CR300, FinePCR, Seoul, Korea). The extracts were centrifuged (4000 rpm, 10 min) and supernatants were collected. Collected supernatants were kept at −70 °C for 2 days and then dried in a freeze-dryer (Labconco freeze-dry/shell freeze system, Labconco Corp., Kansas City, MO, USA). Finally the dried extracts were stored at −20 °C before analysis.

### 3.3. Determination of Physicochemical Property

The moisture content of BG was determined in accordance with the techniques described by the official AOAC method [28]. The pH of BG was measured by a calibrated pH meter (Corning 530, Corning Inc., Corning, NY, USA). The total acid content of BG was analyzed by titration of the sample with 0.1 N NaOH to pH 8.3 and expressed as a percentage of tartaric acid [28]. Reducing sugar content was analyzed by the DNS method [29].

### 3.4. Color Measurements

Color values of BG were performed using a spectrocolorimeter (JS-555 Color Techno System Co. Ltd., Tokyo, Japan) tristimulus color analyzer calibrated with a white porcelain reference plate. The color coordinates of the uniform color space CIE-LAB L*, a*, b* were determined by its reflectance and chromaticity. The L* value indicates brightness ranging from black (L* = 0) to white (L* = 100). The a* value indicates redness ranging from −60 (green) to 60 (red) and the b* value ranges from −60 (blue) to 60 (yellow). Furthermore, in order to confirm the browning development of BG, UV-visible spectra of BG were measured by the absorbance at the range of 200–700 nm using a spectrophotometer (model 1800, Shimadzu, Kyoto, Japan).

### 3.5. Determination of Amino Acids

The amino acids of BG were determined using HP 1100 liquid chromatograph (Hewlett Packard Wilmington, DE, USA) with a variable wavelength detector (VWD HP 1100) operating at 338 nm (excitation = 340 nm). Separation was carried out with a Zorbax Eclipse AAA Rapid Resolution column (150 × 4.6 mm i.d., 5 μm particle size, Agilent Technologies, Palo Alto, CA, USA). A linear gradient profile of mobile phase, comprising 40 mM Na_2_HPO_4_, pH 7.8 (solvent A) and CAN/MeOH/water 45:45:10 (v/v/v) (solvent B), 0% B (0–1.9 min), 0%–57% (1.9–18.1 min), 57%–100% (18.1–18.8 min), 100% (18.8–22.3 min), 100%–0% (22.3–23.2 min) and 0% (23.2–26 min) was applied at a flow rate of 2.0 mL/min. The column was equilibrated for 5 min under initial conditions prior to injection of the next samples. The column temperature was 40 °C. In order to determine the amino acids content of BG, precolumn derivatization with *o*-phthalaldehyde (OPA) was used and 0.5 µL portions were injected into the HPLC system. The data analysis was performed using Chemstation software (Hewlett Packard).

### 3.6. Determination of Antioxidant Contents

The total polyphenol content of BG was determined according to the method described by Arnous *et al.* [30]. Briefly, an aliquot of diluted BG (2.4 mL) was mixed with Folin-Ciocalteu reagent (0.15 mL), followed by the addition of 1 M NaCO_3_ solution (0.45 mL). The reaction was then allowed to proceed for 1 min. The absorbance at 750 nm was measured after 30 min at room temperature in the dark. Total polyphenols content was expressed as milligrams of gallic acid equivalents (GAE).

The total flavonoids content was analyzed according to the method described by Shen *et al.* [31]. Deionized water (2 mL) and 0.5 M NaNO_2_ (0.15 mL) solution were mixed with BG (0.5 mL), and allowed to react at room temperature for 5 min. Then, 0.4 M AlCl_3_∙6H_2_O solution (0.15 mL) was added and the samples were incubated for 5 min before the addition of 1 M NaOH solution (1 mL). The absorbance was measured at 415 nm after 15 min incubation. The total flavonoid content was expressed as rutin equivalents (RE).

### 3.7. Determination of Antioxidant Activity

The free radical scavenging activity of BG, based on the scavenging activity of the stable DPPH (2,2-diphenyl-1-picrylhydrazyl) free radical, was determined using the method described by Brand-Willian *et al.* [32] with slight modifications. A sample of BG (0.2 mL) was added to 0.2 mM DPPH dissolved in ethanol solution (0.5 mL). After incubating the solution at room temperature in the dark for 30 min, the absorbance was measured at 517 nm, and the radical scavenging activity was expressed as percent inhibition:

DPPH inhibition (%) = ([AC − AS]/AC) × 100
(1)

where AC was the absorbance of the control (blank), and AS was the absorbance of the extract.

The free radical scavenging activity of BG, based on the scavenging activity of the stable ABTS•+ [2,2-azinobis-(3-ethylbenzo-thiazoline-6-sulphnate] free radical, was also determined using the method described by Re *et al.* [33] with slight modifications. A mixture of an oxidant (2.45 mM of potassium persulfate) and 7 mM ABTS solution dissolved in 20 mM sodium acetate buffer (pH 4.5) was incubated at room temperature in the dark for 12 to 16 h to create a stable and dark blue-green radical solution. The solution was then diluted with a PBS (phosphate buffered saline) solution to an absorbance of 0.70 ± 0.02 at 734 nm to create the test reagent as a working solution. Then, 0.03 mL of diluted BG was added to 3 mL of the working solution. After incubating the solution at room temperature in the dark for 30 min, the absorbance was measured at 734 nm and the radical scavenging activity was expressed as mmol/L Trolox equivalent per gram of BG.

The antioxidant capacity of BG was determined using the ferric reducing antioxidant power (FRAP) assay described by Benzie and Strain [34] with some modifications. The stock solutions included 300 mmol/L acetate buffer (pH 3.6), 10 mM TPTZ (2,4,6-tripyridyl-*s*-triazine) solution dissolved in 40 mM HCl, and 20 mM FeCl_3_·6H_2_O solution. The working solution was prepared by mixing acetate buffer (25 mL), TPTZ solution (2.5 mL), and 20 mM FeCl_3_·6H_2_O solution (2.5 mL). Black garlic (0.05 mL) was added to the FRAP solution (0.75 mL) and incubated at room temperature in the dark for 30 min. Color changes were then measured at 593 nm and the standard curve was linear between 0 to 200 µM of Trolox. Result was expressed as mmol/L Trolox equivalent per gram of BG.

The reducing power of BG was measured according to the method described by Oyaizu [35] with slight modifications. Briefly, BG (0.1 mL) was mixed with 200 mM sodium phosphate buffer (pH 6.6, 0.25 mL) and 1% potassium ferrocyanide (0.25 mL). The mixture was incubated in a water bath (50 °C) for 20 min and then added to 0.62 M trichloroacetic acid (TCA, 0.2 mL) solution to terminate the reaction. The mixture was then mixed with deionized water (0.2 mL) and 6.17 mM ferric chloride solution (0.5 mL). The absorbance of the resulting solution was measured at 700 nm using deionized water as a blank. The reducing potential of the sample was determined against a standard curve of Trolox (0–200 µM). Results were expressed as mmol/L Trolox equivalent per gram of BG.

### 3.8. Statistical Analysis

All experiments were carried out in triplicate and data was expressed as mean ± standard deviation (SD) using SPSS 17.0 version (SPSS Institute, Chicago, IL, USA). One-way analysis of variance (ANOVA) and Duncan’s Multiple Comparison Test were used to determine the significance of the difference among samples with a significance level of 0.05.

## 4. Conclusions

Black garlic (BG), produced by aging whole bulbs of garlic (*Allium sativum* L.) at 70 °C in 90% relative humidity for 35 days, have shown higher antioxidant properties when compared to raw garlic. Moisture content, reducing sugar and total acidity of BG increased significantly until the 21st day of aging. While lightness and yellowness values of BG decreased dramatically until the 21st day of aging. The maximum absorbance of BG on the 21st and 28 th days were in the visible ranges of around 450 nm, related to the final stage of the Maillard browning reaction. Total polyphenol and total flavonoids contents of BG significantly increased till the 21th day of aging, and changed only slightly thereafter. The antioxidant activities of BG throughout the aging period were consistent with the antioxidant components measured using DPPH, ABTS, FRAP, and reducing power. Therefore, this study of BG might be useful for understanding not only the antioxidant properties of BG, but also its optimum aging conditions for maximized antioxidant properties.
